# Targeting the Cation-Chloride Co-Transporter NKCC1 to Re-Establish GABAergic Inhibition and an Appropriate Excitatory/Inhibitory Balance in Selective Neuronal Circuits: A Novel Approach for the Treatment of Alzheimer’s Disease

**DOI:** 10.3390/brainsci12060783

**Published:** 2022-06-15

**Authors:** Simona Capsoni, Ivan Arisi, Francesca Malerba, Mara D’Onofrio, Antonino Cattaneo, Enrico Cherubini

**Affiliations:** 1 Bio@SNS Laboratory of Biology, Scuola Normale Superiore, 56126 Pisa, Italy; simona.capsoni@sns.it; 2 Section of Physiology, Department of Neuroscience and Rehabilitation, University of Ferrara, 44121 Ferrara, Italy; 3 Fondazione European Brain Research Institute (EBRI) Rita Levi-Montalcini, 00161 Rome, Italy; i.arisi@ebri.it (I.A.); f.malerba@ebri.it (F.M.); mara.donofrio@ebri.it (M.D.)

**Keywords:** depolarizing GABA_A_-mediated neurotransmission, cation-chloride co-transporters, KCC2 dysfunction, Alzheimer’s disease, NGF, AD11 transgenic mice, bumetanide treatment

## Abstract

GABA, the main inhibitory neurotransmitter in the adult brain, depolarizes and excites immature neurons because of an initially higher intracellular chloride concentration [Cl^−^]i due to the delayed expression of the chloride exporter KCC2 at birth. Depolarization-induced calcium rise via NMDA receptors and voltage-dependent calcium channels is instrumental in shaping neuronal circuits and in controlling the excitatory (E)/inhibitory (I) balance in selective brain areas. An E/I imbalance accounts for cognitive impairment observed in several neuropsychiatric disorders. The aim of this review is to summarize recent data on the mechanisms by which alterations of GABAergic signaling alter the E/I balance in cortical and hippocampal neurons in Alzheimer’s disease (AD) and the role of cation-chloride co-transporters in this process. In particular, we discuss the NGF and AD relationship and how mice engineered to express recombinant neutralizing anti-NGF antibodies (AD11 mice), which develop a neurodegenerative pathology reminiscent of that observed in AD patients, exhibit a depolarizing action of GABA due to KCC2 impairment. Treating AD and other forms of dementia with bumetanide, a selective KCC2 antagonist, contributes to re-establishing a proper E/I balance in selective brain areas, leading to amelioration of AD symptoms and the slowing down of disease progression.

## 1. Introduction

γ-Aminobutiric acid (GABA) is the main inhibitory neurotransmitter in the adult mammalian CNS. Released from GABAergic interneurons, GABA binds to two different classes of receptors: GABA_A_ and GABA_B_. While GABA_A_ receptors are integral ion channels, permeable to chloride (Cl^−^) and bicarbonate (HCO3^−^), GABA_B_ receptors are coupled to ion channels via guanine nucleotide-binding proteins and second messengers. GABA_A_ receptors mediate two distinct forms of inhibition: phasic and tonic [1,2]. The first consists of fast inhibitory postsynaptic potentials (IPSPs), regulating point-to-point communication between neurons. In this case, GABA_A_ receptors facing presynaptic release sites are activated by a brief exposure to a high concentration of GABA released by exocytosis of presynaptic vesicles. The second consists of a persistent inhibitory conductance, which is essential for regulating the membrane potential and network excitability. In this case, “ambient” GABA spills out from the synaptic cleft and persistently activates extra synaptic GABA_A_ receptors, which are characterized by high affinity for GABA and relatively poor desensitizing properties [3]. Interestingly, early in postnatal development, GABA depolarizes and excites targeted cells by an outward flux of chloride due to a higher intracellular chloride concentration [Cl^−^]_i_ that results from the differential temporal expression of the cation-chloride co-transporters NKCC1 and KCC2, which are involved in Cl^−^ uptake and extrusion, respectively [4,5]. The low expression of the KCC2 extruder at birth leads to Cl^−^ accumulation inside the neuron via NKCC1 [6].

In adulthood, GABA, via feed-forward and feed-back inhibition, shapes the spatial and temporal profile of pyramidal cell firing, thus exerting a powerful control on neuronal excitability and network oscillations crucial for information processing [7,8]. Although GABAergic interneurons constitute only 15–20% of the total neuronal population, they are nevertheless instrumental in orchestrating the activity of principal cells and to maintain normal oscillatory activity in cortical circuits [7]. Oscillations, which occur at various frequencies supporting different behavioral states and high cognitive tasks, are altered in several neuro-psychiatric diseases including neurodegenerative disorders such as AD [8].

This review will focus on recent data on GABAergic dysfunction in AD, the most common cause of dementia worldwide with serious economic and social burden. Given the projected trends in population ageing and growth, the number of people with AD and other forms of dementia is expected to increase from 57.4 million in 2019 to 152.8 in 2050 [9]. AD research has been for years concentrated on the effects of cholinergic and glutamatergic transmission [10,11,12], while GABAergic transmission has been often neglected [13]. However, the observation that a subset of AD patients are affected by epilepsy, a neurological condition characterized by recurrent seizures caused by an excitatory (E)/inhibitory (I) imbalance [14,15,16], probably secondary to deficits of GABA_A_-mediated inhibitory neurotransmission, suggests the involvement of GABAergic signaling, particularly in cortical and hippocampal networks, which are the most vulnerable brain regions in AD. It is worth mentioning that AD patients carrying the risk factor apolipoprotein E4, a protein involved in lipid and cholesterol transport as well as cell repair and regulating Aβ deposition and neurogenesis, exhibit a deficit in GABAergic signaling. The EEG of patients with moderate AD and young people carrying the *APOE4* variant often exhibit, with respect to age-matched controls, subclinical epileptiform abnormalities, reflecting a state of neuronal hyper-excitability due to a reduced GABA_A_-mediated synaptic inhibition [17,18]. Further evidence in favor of a GABAergic dysfunction in AD is the positive effects on cognitive functions exerted by antiepileptic drugs [17,19,20]. Therefore, in the last years, increasing interest in the role played by GABAergic neurotransmission in AD has been developed in both humans and animal models of the disease.

## 2. Alterations of GABAergic Signaling in Alzheimer’s Disease and in Animal Models

A decreased level of GABA has been found in the cerebrospinal fluid of aged people and AD patients [21]. Using magnetic resonance spectroscopy, a significant reduction of GABA levels has been detected in the parietal region of AD patients [22], an effect that worsens with age [23]. Postmortem studies from AD brains have demonstrated a reduced level of glutamate decarboxylase (GAD) [23], the enzyme responsible for converting glutamate to GABA, an effect associated with a decrease in perisomatic GABAergic terminals, especially in cortical neurons adjacent to amyloid plaques [24,25]. In addition, a reduction of GABAergic interneurons containing somatostatin [22,26], calcium binding protein calretinin [27], calbindin [28], and parvalbumin [29] has been found in brain regions expressing Aβ and tau pathologies as well as neuroinflammation such as the neocortex and the hippocampus [30]. A decrease in somatostatin has been also found in the cerebrospinal fluid of patients with EEG abnormalities and cognitive deficits [31]. A loss of GABA_A_ receptors was also reported in the brain from AD patients [32,33]. Using selective antibodies, α1 and α5 subunits of GABA_A_ receptor were found to be reduced in the hippocampus in a region-specific way, whereas β1, β2, β3, and γ2 subunits were found to be unaffected [34]. While α1 subunits are expressed on synaptic GABA_A_ receptors, α5 are mainly present on extra synaptic receptors, which are involved in tonic inhibition. A significant reduction of GABA_B_ receptors’ immunoreactivity has been also detected in the CA1 region of the hippocampus of AD patients [35]. Furthermore, an age-dependent downregulation of currents evoked by exogenous application of GABA was found in *Xenopus oocytes* transplanted with cell membranes, isolated from human temporal cortex of AD patients and age-matched controls. These currents exhibited a faster desensitization rate and were associated with a reduction of α1 and γ2 subunits and an increased expression of α2, β1, and γ1 subunits of GABA_A_ receptors, suggesting an AD-dependent re-modelling, resulting in atypical GABAergic signaling [36].

Mouse models carrying a specific set of mutations found in humans and mimicking various aspects of AD pathology have been very useful to better understand the molecular and cellular mechanisms underlying this devastating disorder. TgCRND8 mice, over-expressing the mutant human APP at levels approximately 5-fold higher than endogenous murine APP, which exhibit early Aβ deposition, show a loss of GABAergic interneurons already at 6 months [37]. In line with these results, transgenic mice of the APP/PSI models, which overexpress the Swedish family mutated forms of the human amyloid precursor protein and various mutations in presenilin 1, exhibit a significant (50/60%) reduction in the number of GABAergic neurons co-expressing somatostatin (SOM) and neuropeptide Y (NPY), which precedes principal cells loss [38]. This results in an age-dependent increase in neuronal excitability due to E/I imbalance and in spontaneous seizures [39]. The loss of SOM/NPY^+^ GABAergic interneurons, which is associated with the accumulation of Aβ plaques in the extracellular space and to microglial-induced excito-toxicity, was detected also in the entorhinal cortex of six-month-old mice [40]. The E/I imbalance, due to dysfunction or loss of GABAergic interneurons in the dentate gyrus, may account for aging-related memory deficits in AD [7]. It has been also shown that the loss of GABAergic interneurons in the hippocampal hilus of mice carrying the AD risk factor apolipoprotein E4 (*APOE4* knock-in mice) leads to severe age-dependent learning and memory deficits [41,42,43], an effect that can be rescued by transplanting medial ganglionic eminence-derived inhibitory interneuron progenitors into the hilus [43]. Fast spiking, parvalbumin (PV)-containing interneurons pace, via feed-forward inhibition, principal cells, giving rise to gamma oscillations thought to be involved in high cognitive functions [44]. A reduced expression of PV^+^ interneurons has been found in the CA1 region of the hippocampus of 3xTg-AD mice [45] that harbor the mutant genes for amyloid precursor protein (APPS_we_), presenilin 1 (PS1_M146V_) and tau_P301L_.

It is worth mentioning that AD is often associated not only to alterations of GABA_A_-mediated phasic inhibition but also to modifications of the tonic one, probably consequent to the enhanced GABA release from glial cells that express GABA_A_ receptors [46]. In 5XFAD mice, which overexpress human *APP* with three FAD mutations (the Swedish (K670N, M671L), Florida (I716V), and London (V7171) mutations) and human *PSEN1* with two FAD mutations (M146L and L286V), the neuron–glia interaction-mediated enhancement of tonic inhibition would suppress long-term potentiation resulting in memory deficits [47]. The increase in ambient GABA would also reduce ripples oscillations, which are known to be involved in memory consolidation [48]. Other factors contributing to the enhancement of ambient GABA levels include changes in GABA uptake and clearance and expression of GABA_A_ receptor subunits and alterations in GABA transporters. A similar increase in tonic inhibition, mediated by the activation of extra-synaptic α5 subunits containing GABA_A_ receptors, was induced in mice by microinjecting Aβ_1-42_ in the CA1 region of the hippocampus, an area critical for episodic memory. This led to disruption of the E/I balance and cognitive decline [49]. Additionally, reactive astrocytes activated by Aβ have been shown to contribute to memory’s impairment in AD by enhancing ambient GABA with consequent inhibition of synaptic function via GABA_A_ and GABA_B_ receptors [50].

**Figure 1:**
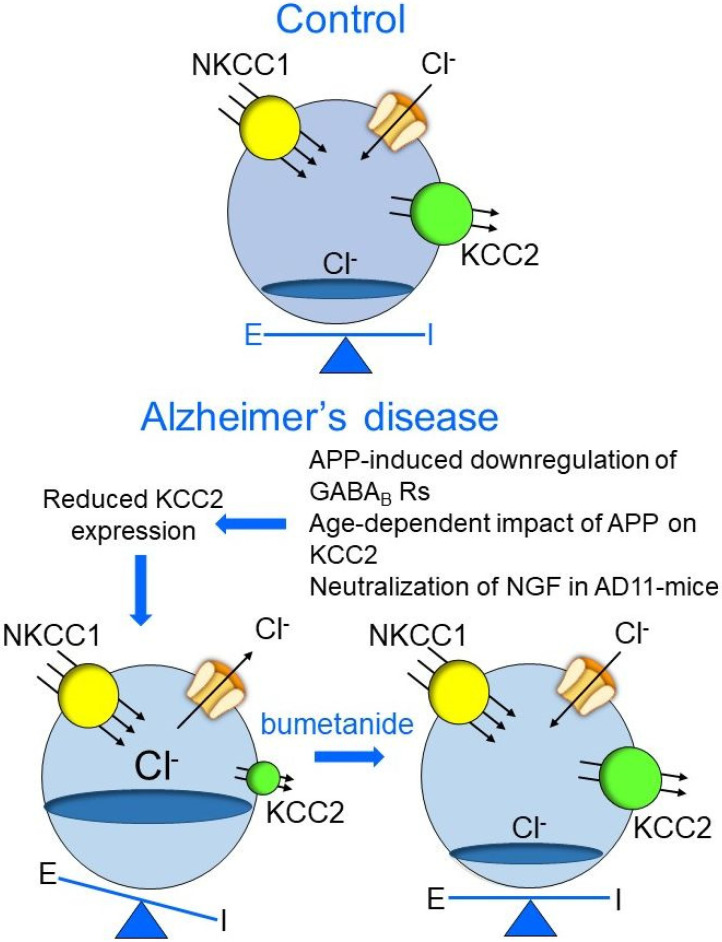
Altered chloride homeostasis in AD. In the adult brain (**top**), the intracellular levels of chloride, are maintained at very low levels by the cation-chloride importer and exporter NKCC1 and KCC2, respectively. GABA binds to its receptor and opens the channel, giving rise to an inward flux of chloride, which inhibits targeted cells by hyperpolarizing the membrane. In this way, it contributes to preserve the appropriate E/I balance in selective neuronal circuits. In AD, the APP-induced downregulation of GABA_B_ Rs, the age-dependent impact of APP on KCC2, or neutralization of NGF in AD11 mice allows GABA to shift from the hyperpolarizing to the depolarizing direction, as in immature neurons, giving rise to an outwardly directed flux of chloride and E/I imbalance with consequent enhancement of network excitability (**down left**), which is an effect that can be rescued by blocking NKCC1 with bumetanide (**down right**).

AD has been found to be associated with a downregulation of GABA_B_ receptors. These receptors act pre-synaptically by inhibiting the release of a variety of neurotransmitters and post-synaptically by generating inhibitory K^+^ currents, which hyperpolarize the membrane and inhibit neuronal firing [51]. As compared to controls, a significant reduction in total protein and mRNA for all GABA_B_ receptor subunits was detected in the hippocampus of six-month-old APP/PS1 mice in the presence of accumulating amyloid plaques. These changes would act by modifying the overall inhibitory tone, leading to behavioral deficits in spatial learning and memory [52]. Since GABA_B_ receptors are present not only on neurons but also on microglia, the possibility that their loss in AD leads to microglia dysfunction and to an inflammatory response cannot be excluded. In addition, using high-resolution microscopy and quantitative approaches, a significant reduction in the surface expression of GABA_B_ receptors in the granule cells of the DG of 12-month-old APP/PS1 mice was found. This was associated to a large amount of senile plaques and a severe impairment of cognitive functions [53]. Furthermore, the genetic loss of APP, which is known to interact with N-terminal Sushi domain of presynaptic GABA_B_ receptors, led to impairment of GABA_B_ receptor-mediated presynaptic inhibition of glutamate release by limiting presynaptic GABA_B_ receptor trafficking and the availability of APP for endosomal processing to Aβ, with consequent enhancement of Aβ production ([54]; Figure 1). Overall, the above-mentioned observations made in humans and in animal models of AD point to GABA and its receptors as key players in AD onset and progression. Evidence has been recently provided that, by physically interacting with the cation-chloride extruder KCC2, G-protein-coupled GABA_B_ receptors contribute to regulate fast synaptic GABA_A_-mediated inhibition though the rapid and sustained change in the ionic driving force for the chloride-permeable GABA_A_ receptors [55]. Therefore, by changing chloride homeostasis and particularly by down regulating the cation-chloride extruder KCC2 in selective brain areas, it is possible in particular conditions to modify the E/I balance leading to an enhanced neuronal excitability consequent to the shift of GABA from the hyperpolarizing to the depolarizing direction as in the immature brain (Figure 1). The polarity of GABAergic neurotransmission is modulated also by the amyloid precursor protein (APP). In in vitro experiments from primary cortical cell cultures expressing human APP, a downregulation of KCC2 but not NKCC1 was detected. This effect was independent of the APP intracellular domain but was correlated with a reduced expression of transcriptional regulator upstream stimulating factor 1 (USF1; [56]; Figure 1). Downregulation of KCC2 led to GABA-induced rise of [Ca^2+^]_i_, an effect that was prevented by the NKCC1 inhibitor bumetanide, indicating that in hAPP-expressing neurons, changes in [Cl^−^]_i_ may contribute to GABA-induced increase in [Ca^2+^]_i_. The impact of APP on KCC2 was age-dependent, as demonstrated by the observation that the absence of APP in 3-month-old APP^−/−^ mice induced an increased expression of KCC2 but in 8-month-old animals a decrease. This was in accordance with the results of Chen et al. [57], indicating that APP can regulate KCC2 protein level by different mechanisms depending on the age examined.

It should be stressed that cation-chloride cotransporters are very labile, and they can be disrupted in several neuropsychiatric disorders [5,58]. Rapid changes in KCC2 function can be elicited in an activity-dependent fashion and involve different post-translational regulation of transporter proteins, including their phosphorylation and regulation at the cell surface [59].

A decrease of KCC2 expression was observed in the hippocampus of mice engineered to express recombinant neutralizing anti-nerve growth factor (NGF) antibodies (AD11 mice), which develop an age-dependent neurodegenerative pathology reminiscent of that observed in AD patients ([60]; Figure 1). Although the mechanisms linking NGF deprivation to KCC2 downregulation remain to be established, this observation has nevertheless opened promising perspectives towards the development of more effective and specific treatments and finalized to restore a proper E/I balance in AD patients. In the following sections, we discuss in detail the relationship between NGF and AD and the AD11 mouse model of AD. We also discuss how treating AD and other forms of dementia with the selective KCC2 antagonist bumetanide can contribute to re-establishing a proper E/I balance in selective brain circuits, leading to amelioration of AD symptoms and the slowing down of AD progression.

## 3. NGF and Alzheimer’s Disease

### 3.1. NGF and the Cholinergic Hypothesis for AD

Memory loss in AD patients has been found to be associated with a selective reduction of basal forebrain cholinergic neurons (BFCNs) [61,62], which provides projections to subcortical and cortical regions, including the neocortex and the hippocampus, which are known to be involved in high cognitive functions such as learning and memory [63]. A significant loss of choline acetyltransferase (ChAT) and acetylcholinesterase (AChE) [64,65], enzymes responsible for the synthesis and breakdown of acetylcholine, respectively, can be observed in the cortex. The progressive deterioration of cholinergic function in AD has led to the so-called “cholinergic hypothesis” [66,67], which gave rise to numerous studies aimed at developing therapeutic tools for delaying or preventing cholinergic neurodegeneration.

The neurotrophin nerve growth factor (NGF), discovered in 1952 by Rita Levi-Montalcini [68], was thought to be involved in the BFCNs loss, among other epigenetic factors.

NGF is primarily involved in the growth as well as in the maintenance, proliferation, and survival of peripheral and central neurons during development and throughout adulthood [69,70,71,72].

The link between NGF and AD was hypothesized a long time ago based on the effects of NGF on BFCNs, which rely on this neurotrophin for their maintenance and survival [73,74]. mRNA encoding for both NGF and NGF protein has been detected in BFCNs targeted areas [75,76,77,78]. This neurotrophin, which is selectively internalized and retrogradely transported via axonal projections from the cortex to BFCNs [79], selectively binds to TrkA and p75NTR, which are expressed on both soma and axons of BFCNs [80,81,82]. Respond to the exogenous administration of NGF with an increase of cholinergic phenotypical markers [73,83]. Most importantly, NGF is able to prevent BFCN death or atrophy following axotomy [74,84,85] or linked to aging [86].

### 3.2. NGF and the Modulation of APP Processing via TrkA and p75 Receptors

Alterations of NGF signaling have been associated not only to cholinergic dysfunction but also to altered processing of APP and tau. Indeed, an aging pathway has been shown to control the TrkA to p75^NTR^ receptor switch and amyloid β (Aβ) production in cells [87]. Moreover, Aβ peptide binds trimers as well as monomers of p75^NTR^ and activates receptor signaling [88]. On one hand, the p75^NTR^ undergoes regulated intramembrane proteolysis by β-secretase, with the β-cleavage sites of APP and p75^NTR^ being highly homologous, releasing p75^ICD^ (intracellular domain) to transmit a signal to the nucleus in analogy to the ICD signal derived by the cleavage of APP [89]. Further direct links between NGF deficits and the activation of the AD amyloidogenic pathway have been demonstrated in cultured hippocampal neurons [90]. The withdrawal of NGF from hippocampal cells induces a rapid increase in APP levels and an overproduction of Aβ peptide, which is followed by apoptotic death. Several studies clearly indicate that extensive tau hyperphosphorylation occur upon NGF deprivation in NGF-differentiated PC12 cells [91,92,93]. In hippocampal cells, a transient tau hyperphosphorylation, temporally related to the activation of the AD-like amyloidogenic pathway, occurs after NGF deprivation [94]. This provides evidence for a mechanism in which a discontinued or limited supply of NGF can activate a pathological pathway of APP and tau processing, triggering downstream apoptotic cell death [95]. This phenomenon is temporally and causally related with an activation of the endogenous amyloidogenic pathway, which was previously reported in hippocampal neurons undergoing cell death upon NGF withdrawal [90]. These in vitro data are reinforced by in vivo data in the AD11 anti-NGF mouse model.

### 3.3. AD11 Anti-NGF Mice: A Link between NGF Deprivation and Alzheimer’s-like Aβ and Tau Neurodegeneration

A further proof of a link between NGF deprivation and AD comes from in vivo studies on AD11 anti-NGF mice. The mice were obtained using the “neuroantibody approach”, according to which non-immune cells can be engineered to express genes encoding for functional immunoglobulins [96,97,98]. Thus, AD11 mice produce the recombinant antibody anti-NGF αD11, which is known to neutralize NGF biological activity [96,97], under the transcriptional control of the human cytomegalovirus promoter. Anti-NGF antibodies, produced in non-lymphoid cells, are expressed in almost all peripheral mouse tissues and in the brain [98]. The phenotype of these AD11 anti-NGF mice resembles the one described in the sporadic human AD, showing synaptic and behavioral deficits. Since the transgenic anti-NGF antibodies are higher in adult than in newborn mice, inhibition of NGF actions effectively occurs in adult animals only [98]. Thus, anti-NGF transgenic mice show an age-dependent neurodegenerative pathology. Indeed, after the second postnatal month, the anti-NGF antibody levels increase by at least three orders of magnitude (up to 200 ng/mL), and neuronal NGF targets, including sympathetic, sensory, and BFCNs, become severely affected [60]. At two to three months of age, mice show a behavioral impairment [98,99] linked to a starting cholinergic deficit and accumulation of hyper-phosphorylated tau in the entorhinal cortex [100]. At six months of age, AD11 mice start to accumulate Aβ intracellularly in hippocampal dystrophic neurites [100,101]. Intracellular accumulation of tau spreads to other cortical regions and to the hippocampus [100]. At this age, AD11 mice show also a further reduction of cholinergic innervation and synaptic plasticity impairment [102,103,104]. Behavioral deficits appear to be more severe, including object recognition and object location deficits [99]. By 15 months of age, Aβ deposition can be found extracellularly [101], accompanied insoluble and hyper-phosphorylated tau and neurofibrillary tangles in cortical and hippocampal neurons [60,101]. They also display cortical neuronal loss, cholinergic deficit in the BFCNs, and spatial memory deficits [60,98,99]. Thus, we conclude that the link between deficits or alterations in the NGF system and AD go well beyond its long-established neurotrophic actions on BFCNs. The failure or imbalance of NGF support could be due to different causes in the overall cascade(s) of events involving NGF bioactivity: (1) decreased NGF synthesis, (2) unbalanced or altered processing, (3) alterations in receptor expression and/or activity or expression ratios, and (4) altered retrograde transport [105]. These events would be “located” upstream of the “amyloid cascade”, which is the central core of AD neurodegeneration [106], and would be part of a negative feedback loop that involves several steps (e.g., links between APP, tau, and axonal transport) [100,107,108].

This conclusion that NGF actions in the brain could be more widespread than envisaged so far calls for an important question about the targets for new targets of NGF actions in the CNS.

### 3.4. Microglia as New Cellular Targets of NGF Actions in the Adult CNS

What are the cellular targets for NGF actions in this negative loop scheme? This question led to investigate whether non-neuronal cells might be NGF targets in the brain. Indeed, it is known that NGF acts not only on neurons but also non-neuronal cells, including astrocytes, oligodendrocytes, microglia, immune system cells, and endothelial blood vessel cells.

Transcriptomics in the AD11 model have shown that neuroinflammation is the earliest phenotypic alteration and is already present at a presymptomatic phase at 1 month of age [100,105,109], anticipating the finding that NGF exerts a broad neuroprotection not only on TrkA expressing neuronal cells but also on the resident immune cells of the brain, i.e., microglia. Indeed, while NGF has been previously reported to be able to modulate microglial cells in culture [110], we specifically found that this neurotrophin expresses functional NGF receptors in vitro and ex vivo with activation of the downstream intracellular signaling [107,108]. Transcriptomic analysis performed after incubation with NGF showed a modulation of motility, phagocytosis, and degradation pathways. These data were confirmed by functional analysis, showing that NGF can increase microglia membrane dynamics and to activate an outward rectifying current that appears to modulate neuronal glutamatergic neurotransmission [111]. In the same study, we focused on the effects on inflammation and Aβ clearance. NGF was able to reduce the expression of inflammatory cytokines and to increase macro-pinocytosis of Aβ toxic soluble fractions. In addition, NGF-treated microglia protected neurons from Aβ-induced loss of spines and chemically-induced LTP deficits [111]. Thus, it can be concluded that NGF exerts a neuroprotective action not only by acting on BFCNs but also by steering microglia towards an homeostatic neuroprotective phenotype [111].

### 3.5. proNGF/NGF Dysmetabolism Is a Trigger for Neurodegeneration

The mechanism of neurodegeneration in AD11 mice is due to the fact that the anti NGF antibody, expressed in the transgenic mice, binds mature NGF with a 2000-fold higher affinity that that for its precursor, proNGF [112]. This creates a complete neutralization of NGF and an excess free proNGF. Thus, the AD11 mice are a model of proNGF to NGF imbalance [113]. In line with this, we demonstrated that (i) inhibiting TrkA [114] or (ii) overexpressing uncleavable proNGF [113] likewise induce a progressive neurodegeneration. Importantly, crossing AD11 mice to a p75-/-knockout rescues the amyloid dependent neurodegeneration [114].

The negative feedback loop linking proNGF/NGF imbalance to the onset of AD neurodegeneration [107,113] received independent support by evidence linking effects of Aβ on proNGF processing by Cuello’s group [115] and by evidence from human AD brains [116,117]. Among the most recent studies linking NGF deprivation to AD are postmortem analysis of AD brains demonstrating high levels of NGF and its precursor proNGF in the neocortex and hippocampus and low levels in the basal forebrain [118,119,120], suggesting a defect in NGF retrograde transport [108].

Moreover, a large body of evidence has shown that not only NGF but also its precursor proNGF, its downstream signaling pathways, as well as the proteases that are able to cut proNGF and to release the mature form of the neurotrophin are involved in the balance between the physiological and pathological phenotype. As other neurotrophins, NGF is translated as a pre-pro-protein [121]. In the trans-Golgi network, proNGF is cleaved by furin to release the mature form of NGF [122,123,124]. Other proteases besides furin can cleave the pro-peptide in the extracellular space [125,126]. ProNGF is not a mere precursor of NGF, but it behaves as an intramolecular chaperon to ensure the proper folding and secretion of the neurotrophin [127]. Interestingly, with respect to the mature form of NGF, proNGF can act in a synergic or opposite way [120,121,122,123,124,125,126,127,128,129,130,131] depending on relative proNGF/NGF ratio and on the type of receptors activated. This would lead to a pro-apoptotic or pro-survival outcome [122,129,131]. NGF and proNGF exert their action through the activation of three distinct receptor subtypes: TrKA, p75NTR, and sortilin. TrkA is a member of tyrosine kinases receptors family; p75NTR is a member of the tumor necrosis factor receptor (TNFR) superfamily [132], and sortilin is a member of the family of Vps10p-domain transmembrane receptors. Sortilin is the specific receptor of proNGF [133,134], while p75^NTR^ and TrKA can be bound by both NGF and proNGF [135]. By interacting with the co-receptor complex of the p75^NTR^ and sortilin, ProNGF can activate the cell death signaling pathway [133]. p75^NTR^ and TrkA receptors are frequently co-expressed and physically interact with each other, thus cooperating in transducing NGF signals [134]. TrkA preferentially binds to the mature form of NGF, while p75^NTR^ promotes cell death in response NGF but only in the absence of TrKA. [136,137,138]. In fact, although both TrkA and p75NTR can individually bind neurotrophins to elicit independent signaling events, the two receptors work together to enhance trophic signaling during development and in the healthy brain by mediating at least 10-fold higher affinity of the TrkA receptor for NGF [139,140,141,142]. On the contrary, in the absence of TrkA or in association to sortilin, activation of p75^NTR^ by proNGF mediates pro-apoptotic signaling in development and/or in neurodegenerative conditions [142,143]. It has been also demonstrated that, in particular conditions, proNGF can bind trkA and p75^NTR^, giving rise to a pro-survival outcome [116,122,144].

It is worth noting that also the proteases engaged in proNGF cleavage and in NGF degradation are involved in NGF metabolism, and their fine tuning contributes to maintaining the physiological conditions in the brain [115]. In synthesis, the mature NGF is produced via a metabolic cascade in which proNGF, zymogens, convertases, and their endogenous regulators are co-secreted from BFCN-targeted neurons in an activity-dependent manner. Upon the extracellular release of these factors, the inactive zymogen plasminogen is cleaved into its active enzyme form, plasmin, by tissue plasminogen activator (tPA) in a process regulated by the tPA inhibitor, neuroserpin. Plasmin is then responsible for the cleavage of the prodomain of proNGF and the release of mature NGF. Moreover, the degradation of the mature NGF is accomplished by the metalloproteases MMP-3 and MMP-9. Their precursors, proMMP-9 and proMMP-3, are regulated by the tissue inhibitor of metalloproteinases-1 (TIMP-1), which cleaves them into their mature forms. According to Cuello’s theory, an alteration of NGF metabolism is able to explain the pathological process in AD, solving the paradox that, in the AD brains, BFCNs degenerate, while the levels of NGF transcripts remain unchanged [111,139,140], and the protein levels of proNGF are greatly elevated [105,118,119,145,146,147]. The NGF dysmetabolism was confirmed both in AD and in down syndrome postmortem brains. [117,148]. Importantly, an involvement of proNGF in some aspects of the AD-like pathogenesis has been confirmed in vivo through the production and characterization of a transgenic mouse over-expressing proNGF, in which learning and memory deficits, increased Aβ-peptide immunoreactivity, and excitatory/inhibitory imbalance were observed [149].

Thus, after more than five decades of studies on NGF and AD, it became evident that the relationship between NGF deprivation and AD cannot be limited to the cholinergic hypothesis. However, in spite the emergence of other pathogenetic mechanisms [150], NGF is still considered a good therapeutic candidate for AD.

## 4. AD11 Anti-NGF Mice: A Link between NGF Deprivation, Dysfunction of GABAergic, and Cholinergic Signaling and AD

Besides neuroinflammation, a decreased expression of ChAT is one of the first signs of degeneration in AD11 brains [98,100]. Consistent with this view, we have provided evidence that, in adult AD11 mice, deficits in cholinergic signaling in the hippocampus, a key structure for learning and memory processes, are mediated by α7 subtypes of nAChRs, which are known to be highly permeable to calcium. Aβ would interfere with α7 nAChRs, which is localized on glutamatergic terminals, thus preventing nicotine-induced enhancement of synaptic efficacy at CA3-CA1 synapses, probably via a perturbed calcium homeostasis and consequent dysfunction of downstream transduction pathways [151]. Furthermore, we found that in both control and NGF-deprived mice, nicotine, via α7- and β2-containing nAChRs, was able at CA3-CA1 synapses to lower the threshold for the induction of long-term potentiation (LTP) and to convert short-term potentiation (STP) or weak LTP into robust LTP, an effect that in AD11 but not in control mice was prevented by GABA_A_ receptor antagonists [152]. The failure of nicotine to boost activity-dependent synaptic plasticity in the presence of bicuculline or gabazine strongly suggests that deficits of cholinergic signaling in AD involve not only glutamatergic but also GABAergic transmission. The latter would be able to rescue nicotine-induced enhancement of synaptic plasticity via re-arrangements of the GABAergic circuit, including the shift of GABA from the hyperpolarizing to the depolarizing direction, which, by removal the magnesium block from NMDA receptors, would facilitate their activation [152]. Thus, in cell-attach recordings that affect neither the resting membrane potential (RPM) nor the [Cl^−^]_i_, from hippocampal slices of 6-month-old AD11 mice (but not age-matched controls), pressure application of the GABA agonist isoguvacine to CA1 principal cells produced a significant increase in firing rate, which is an effect that was prevented by the GABA_A_ receptor antagonist gabazine [153].

**Figure 2:**
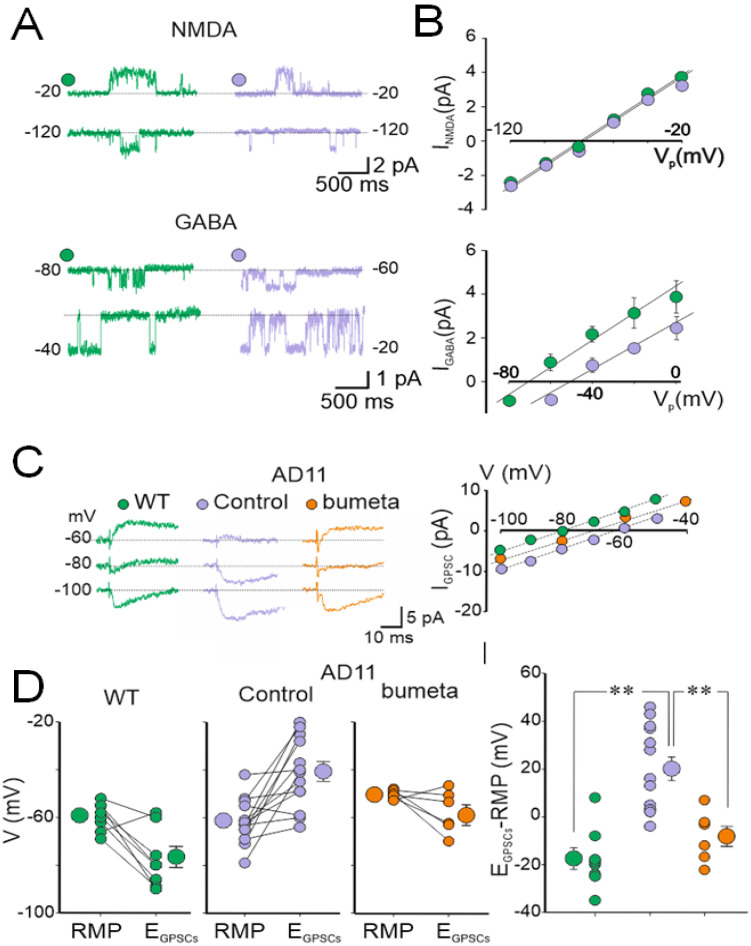
Depolarizing action of GABA in six-month-old AD11 mice. (**A**) Cell-attached recordings of single NMDA and GABA_A_ receptor channels obtained at two different pipette potentials in neurons from WT (green) and AD11 (violet) mice. (**B**) Summary plot of single NMDA (I_NMDA_) and GABA (I_GABA_) currents versus pipette potentials (Vp). Single NMDA currents reversed at −75.3 mV and −76.2 mV in WT and AD11 mice, respectively. Assuming a reversal of NMDA currents equal to 0 mV, we estimated a mean resting membrane potential of −75mV and −76 mV, respectively. In contrast, single GABA_A_ currents reversed at −76 mV and at −56 mV in WT and AD11 mice, respectively. (**C**) Left: Perforated patch recordings of GPSCs evoked in CA3 principal cells by local stimulation of GABAergic interneurons in the presence of DNQX (20 μM) and D-AP-5 (50 μM) at three different holding potentials in hippocampal slices from WT (green), AD11 (violet), and AD11 mice exposed to bumetanide (yellow). Right: Synaptic currents (I_GPSCs_) shown in A are plotted versus membrane potentials (Vm). (**D**) Left: individual RMPs and E_GPSCs_ values in hippocampal slices obtained from WT (green), AD11 (violet), and AD11 exposed to bumetanide (yellow). Larger symbols on the left and right refer to mean ± SEM values. Right: plot of the driving force for GABA_A_ (E_GABA_–RMP) in individual experiments from the three different groups of mice. ** *p* < 0.001 (Modified from [153]).

In addition, while in AD11 mice, GABA-evoked single channel currents reversed at more positive values respect to controls, NMDA-evoked single channel currents, which are used as voltage sensors to measure the resting membrane potentials, reversed at the same membrane potential of control animals (Figure 2A,B). Furthermore, in gramicidin-perforated patch, to preserve the intracellular chloride gradient, the equilibrium potential of GABA_A_-mediated synaptic currents (E_GPSCs_) was more positive in AD11 mice with respect to controls (Figure 2B–D). The driving force (ΔF) equal to (RPM-E_GPSCs_) was positive in AD11 and negative in control mice. These effects could be rescued by the NKCC1 antagonist bumetanide, suggesting that they resulted from accumulation of [Cl^−^]_i_ and the shift of GABA from the hyperpolarizing to the depolarizing direction. These results were associated with a downregulation of the KCC2 cation-chloride exporter protein [153].

**Figure 3:**
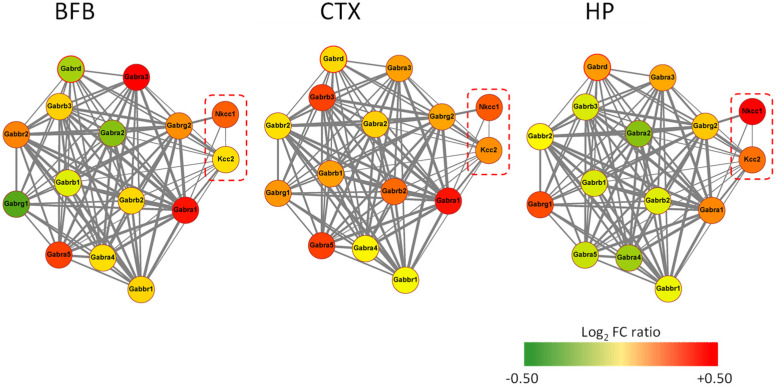
Interaction network in AD11 mice for NKCC1, KCC2, GABA_A_, and GABA_B_ receptor genes. Interaction network extracted from the STRING database (https://string-db.org/, high interaction confidence level) for the NKCC1, KCC2 cation-chloride co-transporter genes (encircled in red), and the GABA_A_, GABA_B_ receptor channel subunits genes in mouse. The node color corresponds to the Log2-fold change-relative expression ratio in 6-month-old AD11 mouse compared to control mouse in three brain areas (basal forebrain, BFB; cortex, CTX; and hippocampus, HP). The thickness of grey edges between nodes corresponds to the interaction reliability provided by STRING.

Interestingly enough, an involvement GABAergic transmission in AD11 mice has also been suggested by the transcriptomic analysis [154,155], which has demonstrated a decreased expression of α2 subunit of GABA_A_ receptor at postnatal day 30 in the cerebral cortex and at P90 also in the hippocampus and basal forebrain region of AD11 [105,150,151], indicating a generally reduced inhibitory drive in the hippocampus and cortex, which is, at this age, a sign of a more general synaptic remodeling. The transcriptomic pattern for the GABAergic system measured in 6-month-old AD11 mice clearly shows a decreased expression of genes encoding for α2, δ, and γ1 subunits of GABA_A_ receptors in the basal forebrain and for α2, α4, and α5 subunits in the hippocampus but not in the cerebral cortex. This effect is coupled to a decreased expression of the gene encoding for KCC2 (Figure 3).

It is worth noting that mice overexpressing proNGF, the precursor of mature NGF, exhibited spontaneous epileptic-like events as the result of a severe E/I imbalance, probably related to a significant reduction of parvalbumin-positive GABAergic interneurons associated with a perineuronal net depletion in the dentate gyrus [149]. However, in this case, the transcriptome analysis unveiled that genes encoding for NKCC1 and KCC2 were not significantly altered.

## 5. Rescuing a Proper GABAergic Signaling for the Treatment of AD by Repurposed Drugs

AD suffers the lack of effective disease-modifying drugs, which are able to directly interfere with the pathogenetic mechanisms of the neurodegenerative condition to reverse or at least stop or significantly slow down the disease trajectory both at the cellular and subcellular level and at the clinical phenotype level. The last decades have seen a large number of failures for randomized clinical trials of pharmaceutical compounds for AD and MCI conditions mainly targeting Aβ. More recent trials targeting tau protein and neuro-inflammation are promising but still ongoing. Complementing the traditional long-term expensive drug development process from the preclinical to clinical trial phases with other approaches, such as drug repurposing and drug repositioning, may be of great help not only to prioritize therapeutic agents for new indications potentially beneficial for patients but also to enlarge the spectrum of applications of drugs and eventually optimize the whole development process as well, with a substantial benefit for pharmaceutical industries [156].

One first example of this approach is represented by two approved drugs, namely camprosate and baclofen, that have been repurposed as potential disease-modifying compounds for Parkinson’s and AD, respectively, and more recently for multiple sclerosis in disease models. As for the AD, based on the fact that camprosate modulates glutamatergic transmission and E/I imbalance, while baclofen activates metabotropic GABA_B_ receptors, the authors show that their synergistic combination, now called PXT864, which targets glutamatergic, GABAergic, and glycinergic systems, protects against Aβ neurotoxicity and alleviates cognitive deficits in a transgenic mouse model of AD [157,158]. A further example is bumetanide, the FDA-approved sulfamyl diuretic, a drug broadly used to treat high blood pressure but also edema associated with heart, liver, or kidney failure. By exerting an inhibitory action on the chloride importer NKCC1, bumetanide is able to restore the inhibitory action of GABA [159]. Bumetanide has been shown to exert positive effects in several neurodevelopmental disorders such as autism, schizophrenia, *fragile X* syndrome, and epilepsy [5,58]. Interestingly, this drug was able to improve cognitive impairments in animal models of down syndrome, a condition known to be associated to an E/I imbalance and related to AD dementia [160].

An impressive study has been recently published on the repurposing of bumetanide for late-onset AD [161]. The scientists’ team, led by Yadong Huang and Marina Sirota at UCSF, extracted the gene expression signature of APOE4 carriers with AD and compared them to non-demented carriers using a temporal lobe transcriptomic dataset. The signature was then compared to the cMap database to find drugs able to normalize the AD APOE4/APOE4 carriers’ transcriptome. To this aim, they ranked the drugs based on their capacity to potentially reverse or flip the AD transcriptomic signature back to control baseline. The significance of fold-change gene shift was computed by a Kolmogorov–Smirnov statistics. Though cMap may not appear a suitable resource, as it is built on cancer cell lines, the strategy was successful since bumetanide ranked 4th out of 1300 compounds as the most effective flipping drug, and it was selected as the top candidate for further investigations because of its safety in long-term use and its positive effects on other brain diseases. The therapeutic potential of bumetanide for AD treatment was validated both in vitro and in vivo and statistically with real-world data. The in vitro validation in iPSC-derived neuronal cells from APOE4/APOE4 subjects confirmed the transcriptomic signatures predicted in silico by the cMap database both for gene expression and pathway analysis. Impressive in vivo validation results were obtained from APOE4/APOE4 knock-in mice and in the same animal models crossed with the J20 mice, which develop amyloid. Following bumetanide treatment, single-cell RNA-Seq showed a reversed transcriptome in many brain cell types together with a reduced amyloid burden, an electrophysiological reversal of LTP, and a recover in spatial memory deficits. Eventually the authors explored the protecting role of bumetanide for AD in a retrospective study on real-world data using electronic health records from two different and independent databases from UCSF University and Mount Sinai NY. The data showed a striking statistically significant lower prevalence of AD in people aged >65 exposed to this drug for indications different from AD: 35% lower in UCSF cohort and even 75% lower in Mount Sinai cohort as compared to controls. In agreement with previously reported data on AD11 mice [153], these results strongly suggest that the shift of GABA action from the hyperpolarizing to the depolarizing direction, consequent to a downregulation of the cation-chloride exporter, KCC2, is responsible for the E/I imbalance observed in AD patients, an effect that could be rescued by blocking the cation-chloride importer NKCC1 with bumetanide.

It is worth noting that, in agreement with [161] in rodent models of AD, it has been recently reported that memory deficiency, consequent to impaired proBDNF/BDNF conversion, which leads to a reduced GABA_A_-mediated neurotransmission in the CA1 hippocampal region associated with reduced KCC2 expression and positive shift of the equilibrium potential for Cl^−^ (E_Cl_^−^), can be rescued by the intracerebroventricular administration of bumetanide, a selective antagonist of the cation-chloride importer NKCC1 [162].

## 6. Conclusions and Future Directions

Cation-chloride co-transporters are key regulators of intracellular Cl^−^ concentration. Their alterations account for several neurodevelopmental and neurodegenerative disorders, including AD. In particular, the shift of GABA from the hyperpolarizing to the depolarizing direction, due to impairment of the cation-chloride KCC2 exporter, is responsible for behavioral deficits observed in the adult AD11 mouse, an animal model of AD, in which neutralization of NGF with selective anti-NGF antibodies leads to a neurodegenerative pathology similar to that observed in AD patients [153]. In recent data obtained by analyzing in two electronic health record databases, a large cohort of patients affected by different neuro-pathologies clearly showed that treatment with bumetanide, a selective antagonist of the chloride importer NKCC1, prevents the development of APOE4-related forms of AD in individuals over the age of 65 years [161]. This suggests that AD is usually associated with a depolarizing action of GABA, probably related to a reduced expression of KCC2, which could be rescued by reducing the expression of NKCC1 with bumetanide. This opens new avenues for developing drugs that, by regulating chloride homeostasis, improve cognitive deficits associated with this devastating disorder. However, in spite of encouraging perspectives, several points need further investigation. It is unclear why NGF, primarily involved in growth, maintenance, proliferation, and survival of peripheral and central neurons during development and throughout adulthood, affects chloride homeostasis. While the cholinergic hypothesis of AD relies on the fact that NGF is involved in the maintenance and survival of thee basal forebrain cholinergic neurons, the primary target of the disease, how this neurotrophin controls cation-chloride co-transporters and the direction of GABAergic signaling remains to be established.

Bumetanide has been shown to have positive effects in a wide range of pathological conditions, but its exact mechanisms of its action are still poorly understood. Although we cannot exclude the possibility that in AD patients, the blood–brain barrier (BBB) is compromised, the effectiveness of bumetanide is usually reduced by its poor pharmacokinetic properties and its low capability to cross the BBB to reach either neuronal or non-neuronal targets [163]. This limitation could be overcome with new derivatives currently under development, which are capable of better permeating the BBB [160]. Alternatively, new therapeutic tools selectively targeting the cation chloride exporter KCC2, which, in contrast to NKCC1, is exclusively expressed on central neurons [164], could be considered. By acting either on KCC2 membrane trafficking or on their intrinsic transport kinetics, these compounds will allow attenuating neuronal [Cl^−^]_i_, reinstating an appropriate Cl^−^ homeostasis in selective brain regions and a proper E/I balance. However, these compounds should not interfere with the well-known KCC2 structural function on dendritic spines, an effect that is independent on its intracellular chloride regulation [165].

Furthermore, in AD11 mice, gene expression experiments have unveiled a downregulation of mRNA encoding KCC2 (but not NKCC1) as well as several GABA_A_ receptor subunits. Therefore, as a matter of speculation, we can foresee the development of a gene therapy approach, whereby genes coding for antibodies delivered via viral vectors to particular brain regions, by selectively silencing the cation-chloride importer NKCC1, will be able to restore GABAergic function by promoting the shift of GABA from the depolarizing to the hyperpolarizing direction with consequent impairment of the E/I balance. This method has been successfully applied to develop two antibodies (mAb 12A12d e scFvA13) targeting the Aβ peptide and the toxic fragment of the tau protein with a high molecular and subcellular precision [166,167].

## Data Availability

All the data supporting the reported results can be found in the literature, appropriately cited here.
